# Total hip arthroplasty with subtrochanteric femoral shortening osteotomy using a monoblock cylindrical cementless stem for severe developmental hip dysplasia (Crowe type III, IV)

**DOI:** 10.1051/sicotj/2024032

**Published:** 2024-09-06

**Authors:** Akio Kanda, Osamu Obayashi, Atsuhiko Mogami, Itaru Morohashi, Muneaki Ishijima

**Affiliations:** 1 Department of Orthopaedic Surgery, Juntendo Shizuoka Hospital Nagaoka 1129, Izunokuni-country 410-2295 Shizuoka Japan; 2 Department of Orthopaedic Surgery, Juntendo University Hongou 3-1-3, Bunkyou ward 113-8431 Tokyo Japan

**Keywords:** Direct lateral approach, Subtrochanteric femoral shortening osteotomy, Cylindrical cementless stem, Severe developmental dysplasia of the hip, Total hip arthroplasty

## Abstract

*Background*: Treatment of patients with Crowe type III and IV dislocated hips is challenging because of the hip deformity in these patients. In addition to the usual total hip replacement, shortening and reduction of the femur are often required. We herein report on our surgical technique using a monoblock cylindrical cementless stem and a direct lateral approach. *Methods*: This study included patients with a diagnosis of severe developmental dysplasia of the hip (Crowe types III and IV) who underwent primary total hip arthroplasty at our hospital from August 2019 to January 2022. Eleven hips of seven patients were treated. All patients underwent horizontal osteotomy using a monoblock cylindrical cementless stem and a direct lateral approach. Complications such as dislocation, infection, and implant dropout were evaluated. In addition, the clinical assessment included the hip range of motion at the last observation and hip function based on the Japanese Orthopaedic Association (JOA) hip score and the Japanese Orthopaedic Association Hip Disease Evaluation Questionnaire (JHEQ). *Results*: The average operation time was 224 min (range, 194–296 min), and the average bleeding amount was 396.1 g (range, 20–1010 g). The main complications were acetabular implant dislocation, postoperative dislocation, intraoperative arterial injury, intraoperative proximal femoral fracture, subsidence of femoral implant. and postoperative pulmonary infarction, which occurred in one patient each. *Conclusion*: Total hip arthroplasty for Crowe type III and IV hips is associated with various surgical technical difficulties because of its anatomical characteristics. While patients with severe osteoporosis are contraindicated, the use of a cylindrical monoblock cementless stem and the direct lateral approach makes it possible to simplify the procedure for shortening the femur and increase the indications for surgery. *Level of evidence*: Therapeutic Level Ⅳ.

## Introduction

Complete hip dislocation occurs in patients with severe developmental dysplasia of the hip. If the patient continues to walk in this state without reducing the dislocation because of lack of treatment or failed open reduction, the center of the femoral head will become elevated, the abduction muscle strength will decrease, and soft tissue contracture will occur [1]. Radiographic findings are characterized by narrowing of the femoral medullary cavity, thinning of the cortical bone, and straightening of the diaphysis [1–6]. In addition to the characteristic X-ray imaging findings, three-dimensional computed tomography shows atrophy of the femoral head, a short femoral neck, hyper-anteversion, posterior movement of the greater trochanter, cranial and dorsal movement of the center of the femoral head, and a narrowed triangular shape of the acetabulum [1, 3–6]. Because of these morphological characteristics, the only treatment option is total hip replacement, which must be combined with femoral shortening osteotomy. We herein discuss the important points in this surgery, such as the approach, selection of the femoral implant, clinical results, and complications such as nerve injury and postoperative dislocation.

## Materials and methods

### Patients

This study included patients with a diagnosis of severe developmental dysplasia of the hip (Crowe types III and IV) who underwent primary total hip arthroplasty at our hospital from August 2019 to January 2022. The patients were followed up for more than 1 year after surgery. We performed eleven cases of total hip arthroplasty (Table 1). Complications such as dislocation, infection, and implant dropout were evaluated. In addition, the clinical assessment included the hip range of motion at the last observation and hip function based on the Japanese Orthopaedic Association (JOA) hip score and the Japanese Orthopaedic Association Hip Disease Evaluation Questionnaire (JHEQ).

**Table 1:** Demographics.

Patient	Age	Sex	Side	Follow-up (months)	Complication	Stem size	Fixation of osteotomy	JOA (preop)	JOA (preop)%	JOA (postop)	JOA (postop)%	JHEQ (preop)	JHEQ (preop)%	JHEQ (postop)	JHEQ (postop)%
1	60	F	R	36	–	11	Bone	71	71	90	90	47	53.4	62	70.5
2	76	F	L	32	Displacement of the cup	11	GTR	42	42	88	88	61	69.3	50	56.8
3	73	F	R	42	Dislocation	12	GTR	59	59	81	81	24	27.3	27	30.7
4	62	F	L	30	–	13	Bone	38	38	96	96	47	53.4	72	81.8
5	75	F	L	36	–	14	Bone	77	77	79	79	55	62.5	66	75.0
6	62	F	R	26	–	13	Bone	40	40	96	96	47	53.4	72	81.8
7	76	F	R	36	–	14	Bone	79	79	81	81	55	62.5	59	67.0
8	67	F	L	38	Arterial injury pulmonary thrombo embolism	11	GTR	53	53	90	90	59	67.0	51	58.0
9	51	F	R	35	–	11	GTR	61	61	59	59	64	72.7	62	70.5
10	73	F	R	24	Subsidence of femoral implant	14	GTR	8	8	59	59	11	12.5	50	56.8
11	84	F	R	12	–	14	Bone	44	44	80	80	11	12.5	50	56.8
				31.5	–			52	52	81.7	81.7	43.7	49.7	58.3	66.3

### Surgical method

**Figure 1:**
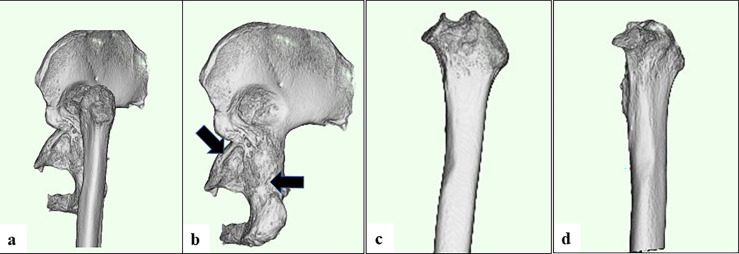
The femoral-side implant. (a) First placed according to the femoral anteversion, and the compatibility of the proximal femoral shape with the implant was confirmed. (b) Placement was then performed with appropriate anteversion reduction according to the distal femur, and the compatibility of the distal stem was confirmed.

A two-dimensional drawing can be performed using the X-ray image of the hip joint, but because the femoral head is located on the cranial and dorsal sides of the original position [1–3, 5], so the actual femoral head displacement distance is longer. However, the ZedHip three-dimensional template (LEXI, Tokyo, Japan) calculates the head displacement distance in three dimensions, thus approximating the actual head displacement distance. Because there is a risk of sciatic nerve paralysis with leg lengthening of ≥ 4 cm [3–9], the length obtained by subtracting 4 cm from the planned reduction length was defined as the pned femoral osteotomy length in this study. The implant choice is important because Crowe type IV hips have characteristic anatomical features. The acetabular implant in this study was sized to ensure bone coverage in the anteroposterior direction at the height of the original acetabulum with an abduction angle of 40° and an anteversion angle of 15°. In this study, the femoral-side implant was first placed according to the femoral anteversion, and the compatibility of the proximal femoral shape with the implant was confirmed (Fig. 1a). Placement was then performed with appropriate anteversion reduction according to the distal femur, and the compatibility of the distal stem was confirmed (Fig. 1b). Based on the data obtained from this process, it was possible to reproduce and evaluate the postoperative internal rotation change in the osteotomy part before the operation.

The posterior approach is often used; however, because sciatic nerve palsy due to the use of the posterior approach and a retractor has been reported [2, 10], we used the direct lateral approach in the lateral decubitus position.

We used a hemispherical porous cementless cup (G7^®^ OsseoTi shell; Zimmer Biomet). This implant has a strong scratch porous and is expected to provide adequate initial fixation. However, screw fixation was added because the bony coating around the cup was often insufficient.

**Figure 2:**
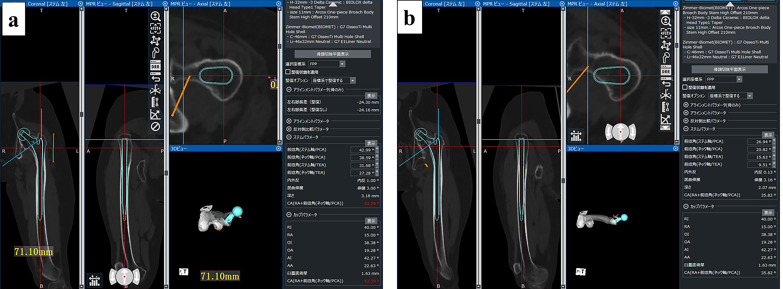
Fixation method of osteotomy. (a) Cable fixation was added to the excised bone fragment. (b) Plate fixation was further added if there was concern about the adequacy of fixation.

We used the horizontal osteotomy method because of the ease of adjusting the amount of shortening and the amount of version correction. Cable fixation was added to the excised bone fragment (Fig. 2a), and plate fixation was further added if there was concern about the adequacy of fixation (Fig. 2b).

We used cementless, slim, monoblock, distal-cylindrical stems (Arcos^®^ One-Piece Broach Body Stems; Zimmer Biomet). The actual surgical procedure was as follows. First, after reaming and rasping along the original broach consistent with the excessive anteversion, shortening bone cutting was performed by the transverse method. The trial implant was then inserted while the proximal bone fragments were rotated to the appropriate anteversion angle. After confirming the stability of the trial implant against reduction and dislocation, the femoral implant was inserted. The contact of the osteotomy was checked; if the distal bone was stable, the excised bone was wrapped with wiring for fixation, and if rotational instability was observed, a greater trochanteric fixation cable was used (GTR cable system; Zimmer Biomet) (Fig. 2b).

Statistical analyses were performed using ystat2000.xls. The Wilcoxon t-test was used for the statistical analyses, and the range of motion of the hip joint, JOA hip score, and JHEQ score were statistically analyzed before and after surgery.

## Results

The average operation time was 224 min (range, 194–296 min), and the average bleeding amount was 396.1 g (range, 20–1010 g). The main complications were acetabular implant dislocation, postoperative dislocation, an intraoperative proximal femoral fracture and subsidence of femoral implant, and intraoperative arterial injury and postoperative pulmonary infarction, which occurred in one patient each. The JOA score significantly improved from 52.0 ± 6.2 to 81.7 ± 3.8 points (52–81.7%). In contrast, the patient-oriented JHEQ score improved from 43.7 ± 20.7 points preoperatively to 58.3 ± 13.2 points (49.7–66.3%) postoperatively, but the difference was not significant (Table 1).

## Discussion

**Figure 3:**
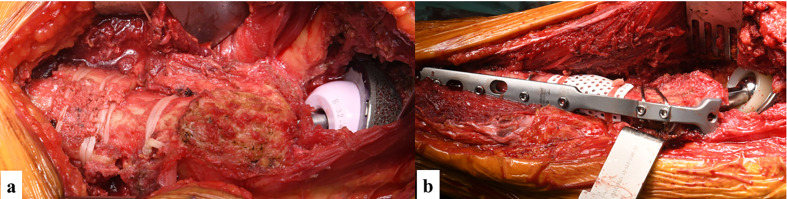
(a) The fibers of the gluteus medius muscle are oriented in a more anteroposterior direction than usual. (b) This lateral run of the gluteus medius muscle becomes vertical when the original acetabular place is installed, and the gluteus medius muscle strength is increased.

**Figure 4:**
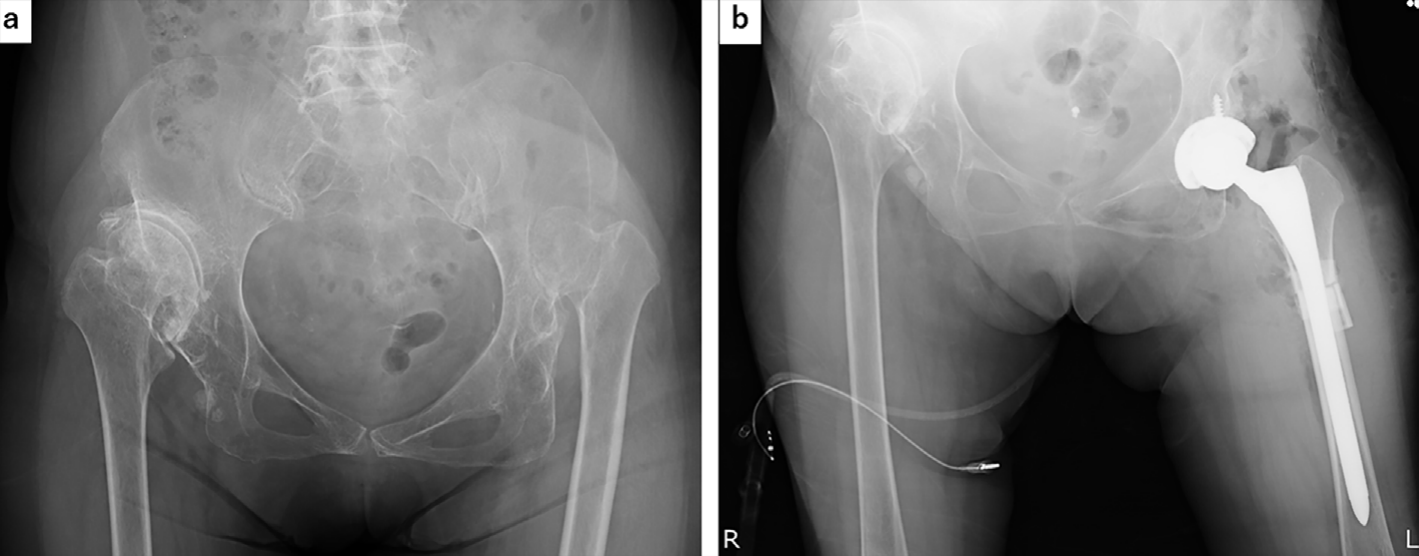
(a) Preoperative X-ray image. Bilateral developmental dysplasia of the hip. (b) Postoperative X-ray image. If the shortened femoral bone fragment is fixed to the osteotomy with a cable, it can sufficiently withstand full weight-bearing walking immediate weight bearing.

In patients with dislocated Crowe type IV hips, the anteversion of the femoral neck is large and the femoral head is displaced dorsally. Therefore, it is easy to obtain a good operative field of view for the femoral head and acetabulum using the direct lateral approach, which is an anterior lateral approach. When unfolded, the fibers of the gluteus medius muscle are oriented in a more anteroposterior direction than usual (Fig. 3a). This lateral trajectory of the gluteus medius muscle becomes vertical when anatomic acetabular height is restored, and the gluteus medius muscle strength is increased (Fig. 3b). Therefore, the position of the acetabular cup is the position of the original acetabulum. Placing the prosthesis restores the proper hip biomechanics and center of rotation because of the reduced hip force and increased abductor moment arm [6, 8, 11]. Furthermore, when the implant on the acetabular side is closer to the original acetabulum, the incidence of loosening decreases [3, 5, 6, 11]. The femur in patients with Crowe type III and IV hips is characterized by atrophy of the femoral head, an increased anterior anteversion angle, a narrow medullary cavity, and a straight diaphysis [1–6, 8, 12]. The amount of shortening when performing shortening osteotomy should take into account the stiffness of the pelvic tilt, lumbar spine deformity, hip range of motion, and soft tissue tension [13]. The amount of movement of the center of the head is measured from the preoperative three-dimensional plan, and the amount of shortening is set so that the leg lengthening is within 4 cm to prevent nerve damage. In addition, if reduction is not possible because of contracture during surgery, shortening osteotomy is added. Bone-cutting methods include the transverse method, oblique method, double-chevron method, step-cut method, and sigmoid method, and each technique has various advantages and disadvantages [2, 5, 6, 8, 9, 14]. The double-chevron method provides rotational stability and a large contact area of the bone; thus, even if a short stem is used, fixation of the osteotomy can be obtained with good bone fusion [14]. Similarly, the step-cut method and oblique method provide good rotational stability, and bone fusion is easy to obtain [6, 9, 15, 16]. These osteotomy methods have evolved to address the shortcomings of transverse osteotomy. However, these methods are complex and technically difficult [3, 5, 8, 13, 14]. Long stems, square stems, plate fixation, and bone grafting may be required to compensate for the indicated weakness of rotational stability [1, 2, 8, 14]. In a recent report, the excised femoral bone fragment was fixed to the osteotomy with a cable, and sufficient rotational stability, a shortened surgical time, and early bone fusion were reported [17]. Concerning the stability of the osteotomy, which is a clinical concern, Muratli et al. [18] reported that the stem shape of cementless stems is irrelevant; however, Tuncay et al. [19] reported that a cylindrical stem rather than a square stem has excellent stability. When using a cemented stem, delayed bone union and poor union may reportedly occur by cement entering the osteotomy [1, 4, 6]. Therefore, cemented stems are often used when cementless stems cannot be used in a narrow medullary cavity. The most recent reports of cementless stems describe modular distal cylindrical stems. Good results have been reported with the general use of these stems because the appropriate size can be selected for both proximal and distal sites, and the anteversion of the stem can be freely adjusted [5, 17, 20–22]. However, the modular type stem is structurally thicker, and the thick stem junction may not fit into the medullary cavity [8]. In contrast, the monoblock type stem has a structurally thinner stem design, allowing it to more easily fit in the lesser trochanter level position [8]. The advantage of this stem is that it is a linear cylindrical stem that allows for reaming and proximal rasping of the distal medullary cavity before femoral osteotomy. Rasping and reaming after femoral osteotomy result in instability, which increases the technical difficulty. Such a method is possible because of the straight distal cylindrical stem; the axis of rotation is aligned with the straight distal femur, and it is possible to reduce the twist. Therefore, the implant itself does not require a twist-reducing function or a modular neck. The cylindrical stem has sufficient contact area with the cortical bone for initial fixation of the distal portion, and it is expected to provide initial fixation and rotational stability through good scratch fit and effective fins as well as early bone healing of the osteotomy. If the shortened femoral bone fragment is fixed to the osteotomy with a cable, it can sufficiently withstand full weight-bearing walking from the immediate weight-bearing (Fig. 4) [17]. According to the reports of each study, the postoperative dislocation rate ranged from 0% to 19%, the incidence of neuropathy ranged from 0% to 14%, the intraoperative fracture rate ranged from 0% to 24%, the union failure of the osteotomy ranged from 0% to 8%, and clinical results showed improvement in all studies [9]. Although only eleven hips were treated at our hospital in the present study, we believe that the results are comparable. An important complication of this surgery is sciatic nerve palsy. In patients undergoing primary total hip arthroplasty, reported risk factors for postoperative nerve palsy include complete hip dislocation, femoral head osteonecrosis, an underweight status, and a low body mass index; however, leg lengthening is not a reported risk factor [22]. In other studies, the appearance of nerve paralysis in patients who have undergone primary total hip arthroplasty for a dislocated hip joint such as a Crowe type IV hip is about 4 times higher than that after general total hip arthroplasty [21], and many reports have indicated that the lower leg lengthening should be ≤ 4 cm [2, 4, 8, 9, 11]. However, there is a risk of paralysis even if the preoperative condition is ≤ 4 cm, such as in patients with a history of Schanz osteotomy or with the development of secondary arthropathy in the hip bone or femur [23, 24]. In addition, Eggli et al. [10] reported that sciatic nerve palsy and leg extension were not related and were caused by mechanical injury due to the difficulty of the procedure [10]. Moreover, Dunn and Hess [25] stated that direct injury by the retractor caused sciatic nerve injury when surgery was performed using the posterior approach. Kong et al. [23] stated that although the posterior approach was used, many direct injuries and nerve injuries occurred during dislocation reduction, especially in patients who had previously undergone surgery. When using the posterior approach, the sciatic nerve was stretched and damaged because of the towing of the lower limbs in the hip flexion position [23, 24]. In this respect, the direct lateral approach is less likely to cause sciatic nerve palsy because the leg is pulled during a reduction in an extended rather than flexed position, thereby reducing sciatic nerve stretch [24]. It is therefore important to reduce the amount of leg extension to ≤ 4 cm, perform protective surgical operations, and use the direct lateral approach. Another complication of this surgery is postoperative dislocation. Immediately after surgery, the gluteus medius muscle is weak and the joint force is small, making dislocation likely to occur. In this study, in one case, intraoperative fracture for severe osteoporosis and an enlarged medullary cavity caused the subsidence of the femoral implant. The patient was replaced with a cemented stem 4 months postoperatively. The present method was found to be contraindicated in severe osteoporosis. In the other 10 cases, the gait was more than walking with a cane and the gait was stable. This study had two main limitations. First, the number of cases was small. Second, the follow-up period was short. Careful and longer follow-up is required in the future. Total hip arthroplasty for Crowe type III and IV hips is associated with various surgical technical difficulties because of its anatomical characteristics. While patients with severe osteoporosis are contraindicated, the use of a cylindrical monoblock cementless stem and the direct lateral approach makes it possible to simplify the procedure for shortening the femur and increase the indications for surgery.

## Data Availability

The data during the current study are available from the Corresponding author on reasonable request.
